# Items Worth Reading

**Published:** 1891-08

**Authors:** 


### Items Worth Reading.

"One of the worst conditions that we meet with in the various aspects of pyorrhœa alveolaris is where, through the ravages of the disease, death of the pulp has ensued and there is added to the original septic matter the pus from the broken down pulp. This condition generally takes place without any warning to the patient; in fact, it is impossible to learn at what time the death of the pulp takes place." DR. M. L. RHEIN.

